# Adaptive Switching Redundant-Mode Multi-Core System for Photovoltaic Power Generation

**DOI:** 10.3390/s24237561

**Published:** 2024-11-27

**Authors:** Liang Liu, Xige Zhang, Jiahui Zhou, Kai Niu, Zixuan Guo, Yawen Zhao, Meng Zhang

**Affiliations:** 1 Smartchip Microelectronics Technology Co., Ltd., Beijing 100000, China; liuliang@sgchip.sgcc.com.cn (L.L.); zhangxige@sgchip.sgcc.com.cn (X.Z.); zhoujiahui@sgchip.sgcc.com.cn (J.Z.); 2 School of Computer Science, Northwestern Polytechnical University, Xi’an 710000, China; 2020264838@mail.nwpu.edu.cn (K.N.); 2023262867@mail.nwpu.edu.cn (Z.G.); yawenzhao@mail.nwpu.edu.cn (Y.Z.)

**Keywords:** adaptive switching, reliability, TMR, DMR, MPPT

## Abstract

As maximum power point tracking (MPPT) algorithms have developed towards multi-task intelligent computing, processors in photovoltaic power generation control systems must be capable of achieving a higher performance. However, the challenges posed by the complex environment of photovoltaic fields with regard to processor reliability cannot be overlooked. To address these issues, we proposed a novel approach. Our approach uses error rate and performance as switching metrics and performs joint statistics to achieve efficient adaptive switching. Based on this, our work designed a redundancy-mode switchable three-core processor system to balance performance and reliability. Additionally, by analyzing the relationship between performance and reliability, we proposed optimization methods to improve reliability while ensuring a high performance was maintained. Finally, we designed an error injection method and verified the system’s reliability by analyzing the error rate probability model in different scenarios. The results of the analysis show that compared with the traditional MPPT controller, the redundancy mode switchable multi-core processor system proposed in this paper exhibits a reliability approximately 5.58 times that of a non-fault-tolerant system. Furthermore, leveraging the feature of module switching, the system’s performance has been enhanced by 26% compared to a highly reliable triple modular redundancy systems, significantly improving the system’s reliability while ensuring a good performance is maintained.

## 1. Introduction

Multi-task intelligent computing requires processors with higher performance for multitasking control and computation because of its task complexity and parallelism. In photovoltaic (PV) power generation control systems, the processor is responsible for the maximum power point tracking (MPPT) algorithm and managing the entire system [1,2,3]. With the advancement and updates in MPPT algorithm research, modern MPPT algorithms are utilizing intelligent computing with increasing frequency. However, in PV fields, abundant solar radiation and temperature fluctuations can lead to signal bit flips in processors, resulting in soft errors [4]. This presents new challenges with regard to the reliability of processors. Therefore, a processor that balances both performance and reliability is essential in the field of PV.

Soft errors occur when external radiation or electrical interference causes temporary faults in the system. These errors can randomly alter data bits, leading to potential system failures or data corruption [5]. Redundant computing is a useful strategy for mitigating soft errors. Redundant computing employs many central processing units (CPUs) to execute the same program and uses a voting mechanism to determine the most accurate outcome. This approach allows for quick error detection and ensures system stability by tolerating faults. However, the use of redundant computation might impede the performance enhancement attained by parallelism in multi-core systems [6]. Grid-tied PV systems must continuously compute the maximum power point to ensure optimal energy production.

**Figure 1:**
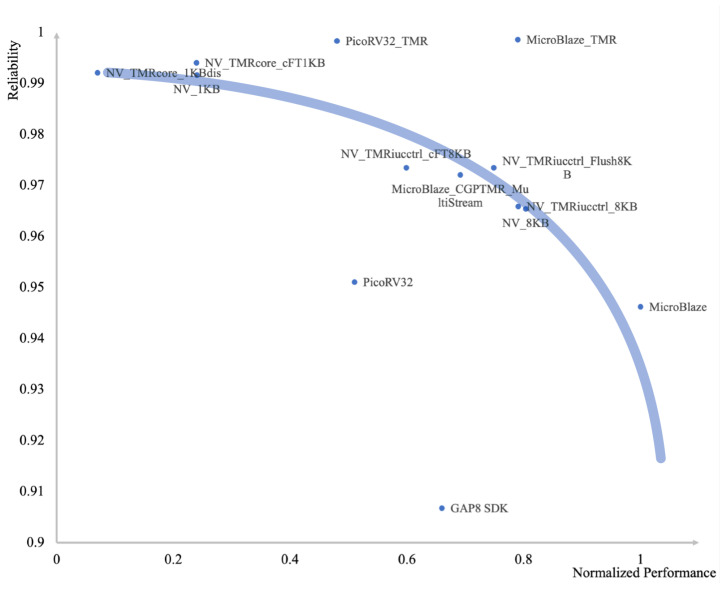
The relationship between performance and reliability.

We discovered performance–reliability curves, as shown in Figure 1, through an extensive survey of multi-core systems [7,8,9,10,11]. The reliability metric used is 1−error_rate. The performance (the product of the maximum frequency and a 32 × 32 matrix multiplication benchmark cycle count) is normalized. Increased reliability leads to a large decrease in performance. From this observation, it is evident that maintaining system reliability within a narrow range of performance deterioration is crucial to guarantee that the performance adequately supports the regular operation of PV controller systems. Hence, it is essential to use appropriate strategies in order to sustain performance while attaining a certain degree of reliability.

Several studies have attempted to achieve an appropriate balance between performance and reliability by employing adaptive switching between performance mode and reliability mode to accommodate varying circumstances [12,13,14]. Baharvand et al. [15] introduced Adaptive Reliability Management by On-the-fly Redundancy in Multi-core Embedded (ARMOR) Processors, ingeniously leveraging an adaptive method to assign cores to perform critical tasks while balancing reliability and limited resources. Some of these studies have explored implementing switching at the application level, which involves inserting switching instructions or detecting error rates to dynamically switch between performance mode and reliability mode [16]. However, this approach does not fully utilize the hardware’s capability to detect errors in real time. Allocating redundant threads to applications through software at the operating system level and dynamically building DMR and TMR fault-tolerant modes is another potential approach. Asghari et al. [17] spotlighted a software-based method by utilizing a task-level redundancy in the operating system to meet the reliability requirements dynamically. A multiplexed redundant execution (MRE) thread-based method is delineated in [18], comparing the outputs of the leading thread and trailing thread to detect errors. However, these methods cannot effectively address transient hardware errors such as soft errors [19]. Therefore, implementing adaptive switching at the hardware level is more suitable for solving the problem of soft errors. For example, in the adaptive lock-step quad-core system [20], the system switches between DMR and TMR systems by monitoring the number of errors occurring within a specific period. However, this solution does not consider the system’s performance requirements.

It is common knowledge that adaptive switching methods can be used to strike a balance between reliability and performance [21]. Nevertheless, without offering thorough justifications for the switching metrics, the majority of efforts have concentrated on the development of redundant processing structures and the use of mode switching. As a result, their performance may be excellent in terms of switching and dependability but not in terms of adapting to various situations. Furthermore, employing a single metric for all switching purposes may result in frequent switches, which in complicated cases might affect productivity. Since PV controller systems are ultimately application-oriented, we are primarily concerned with creating dynamic switching techniques that are adaptive and catered to the demands of certain scenarios.

The lock-step [22] and voting methods must be incorporated into the multi-core framework in order to facilitate N-modular redundant (NMR) calculations and address the previously described concerns. Lock-step is an error detection technique that uses multiple identical cores to execute the same task synchronously and is connected to a verification module. When the verification module detects a discrepancy, it indicates that a system fault has occurred. Voting methods are used in systems with two or more core lock-step systems, where the output results of the cores are compared to vote out the faulty core. For example, in a three-core lock-step system, if the output results of core 0 and core 1 differ from the remaining core 2, this proves that core 2 is at fault. Using these two methods will guarantee the system’s reliability. Additionally, in order to accommodate various application scenarios and satisfy the necessary performance and reliability standards, a switching mechanism that can transition between different modes of the system should be devised. Implementing pertinent technologies in high-performance mode will ensure that programs are executed accurately. This article suggests a three-core adaptive switching system based on TMR and DMR structures to meet these needs. The primary contributions of this system are as follows:

- A hardware-based dynamic switching mechanism that analyzes the performance and error rate of the system in the current scenario is used to adaptively transition between different scenarios. This mechanism utilizes the hardware architecture to statistically analyze the error rate and performance, which serve as the switching metrics.
- A redundancy-mode switchable multi-core(RMSM) processor system that can switch between three modes: balanced (using DMR and single-core parallelism), high-reliability (based on TMR), and high-performance (using three-core parallelism) is designed and constructed.
- Checkpoint backup, pipeline rollback techniques, and fault isolation mechanisms are optimized to rectify errors in processors that go beyond redundant fault tolerance while also enhancing system reliability in high-performance modes.
- A soft error probability model is constructed based on the mechanism and patterns of soft error occurrences, and soft error injection techniques are implemented to validate system reliability, which is an efficient method for validating redundant systems.

This article is organized as follows: Section 2 analyzes the challenges associated with designing a redundancy-mode switchable multi-core(RMSM) processor system and effectively evaluating the system’s performance and reliability. Section 3 indicates the detailed strategy of dynamic switching. The proposed RMSM processor hardware architecture and associated optimization approaches are illustrated in detail in Section 4. Section 5 provides evaluations and comparisons. This paper is concluded in Section 6.

## 2. Analysis

### 2.1. Challenge 1: Selection of Switching Indicators

**Figure 2:**
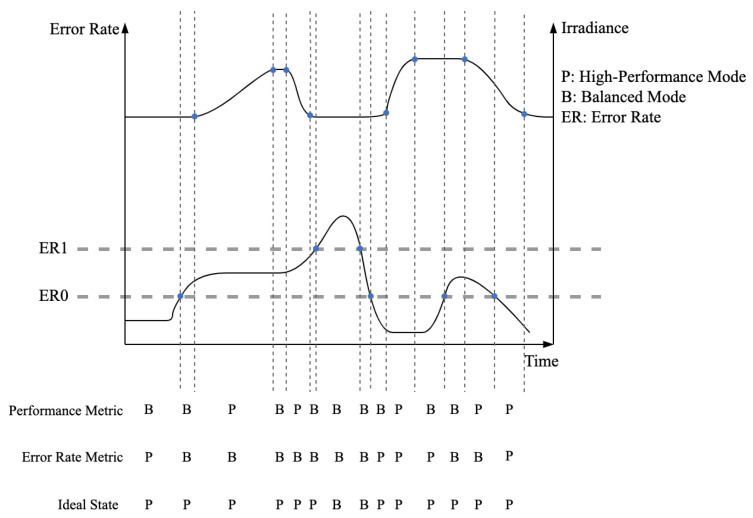
Reliability simulation of real scenarios. Impact of switching metrics on mode transitions in PV controllers.

There is no obvious way to distinguish changes in the complex scenarios that make up the PV controller system’s real working environment. It is not feasible to switch the redundant mode directly in accordance with scene switching. In order to guarantee that at least one mode among the several modes will satisfy the scenario’s performance and reliability criteria, the scenario requirements must be precisely studied and quantified so the system can be considered completely applicable to the application scenario. The circumstances within the same scene are always shifting. The hypothetical situation is shown in Figure 2; the modes involved are high-performance and balanced modes. The performance requirements for PV controllers increase when the rate of change in irradiance is relatively rapid. Therefore, the slope of irradiance change is used as a quantitative metric for PV controllers’ performance. When using performance as the sole switching metric, the mode is switched seven times. When using the error rate threshold as the sole switching metric, the frequent fluctuations of error rates in scenarios lead to frequent mode switching with four switches. Utilizing these two metrics as independent sole switching metrics results in high-frequency mode switching, leading to low reliability and affecting the performance of PV controllers. Ideally, we aim for the error rate threshold range and performance to serve jointly as switching metrics. In this ideal state, the number of switches would be reduced to two, achieving a balance between performance and reliability.

### 2.2. Challenge 2: Balance Between Reliability and Overhead

In addition to degrading performance, redundant processing increases overhead. Hardware overhead is higher for multi-core processor systems that offer redundant computation than for conventional multi-core systems. These include the following:

- Output comparison is necessary for TMR and DMR designs. TMR is capable of identifying the origin of a mistake by means of a majority voting circuit. It then proceeds to rectify the problem by moving forward with the recovery process, guided by the outcome of the voting circuits. However, DMR has the capability to detect the outcome but lacks the ability to accurately identify the proper outcome [23].
- Mistakes like branch prediction failures, aside from the impact of soft errors, cannot be resolved by redundant computations. For error recovery, these mistakes need extra rollback technology [24].
- The suggested adaptive dynamic switching system aims to address the issue of soft errors by alternating between different modes. Lock-stepping several CPUs imposes requirements on the structure.

Obviously, the continuous pursuit of computing reliability and accuracy will lead to a continuous increase in hardware overhead.

### 2.3. Challenge 3: A Method of Efficient Evaluation

During the design process, the system needs to be verified multiple times to achieve the optimal needs of the design space. The simulator conducts modeling and evaluation through different abstraction levels, such as instruction flow or timing, and the verification speed is slow. There are methods that can be used to quickly verify the performance of the system through analytical models. Still, analytical models can only find theoretical optimal points based on modeling and cannot effectively guide actual design. The traditional simulator verification method tests and verifies the performance and reliability of the system after the complete actual system design, and then feeds the results back to the system design. This full-cycle, iterative process is inefficient and costly. To explore the design space efficiently, a more efficient verification method is needed to simultaneously perform an evaluation and provide feedback during the design phase.

## 3. Dynamic Switching Strategy

### 3.1. Switching Metrics Based on Error Rate and Performance

In order to achieve a balance between performance and reliability, while also maintaining appropriate costs for PV controller systems, it is crucial to select quantifiable metrics for adaptation. In this research, we have chosen two metrics for evaluation: the statistical analysis of soft error rates throughout a specific time interval and performance requirements determined by operational scenarios. The former employs adaptive fault-tolerant configuration registers to guarantee the reliability of the system, enabling a customizable statistical analysis of soft error rates within defined time intervals. This fulfills the requirements for adaptability in various application settings. The latter utilizes performance counters to monitor the real-time performance of the processor. The software level of the processor determines its performance requirements by detecting system performance monitoring registers and enables the switch to redundant modes. Redundant mode switching is not exclusively determined by hardware soft error rates when the application’s fault tolerance needs are low or the software already has a certain level of inherent fault tolerance. Alternatively, the performance sampling function is utilized to sample the present situation. When the mean value of the performance sampling counter is within a designated range, the system transitions to the associated redundant mode according to its performance criteria. On the other hand, if the application requires greater fault tolerance, the mode switching is not based on performance sampling counters but is instead determined dynamically by the hardware’s soft error fault detection rate.

### 3.2. Joint Statistical Dynamic Switching Mechanism Based on Error Rate and Performance

**Figure 3:**
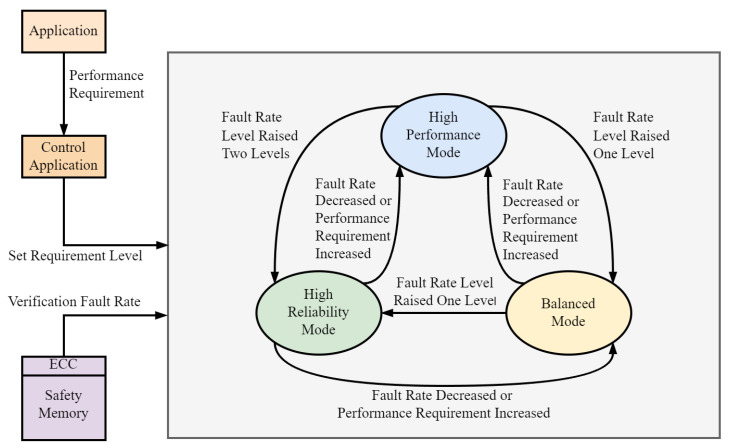
Adaptive mode switching based on performance requirements and fault rate levels.

This paper proposes a joint statistical dynamic switching mechanism based on error rate and performance, focused on error rate statistics, and built upon the foundation of software-controlled mode switching, as shown in Figure 3.

**Figure 4:**
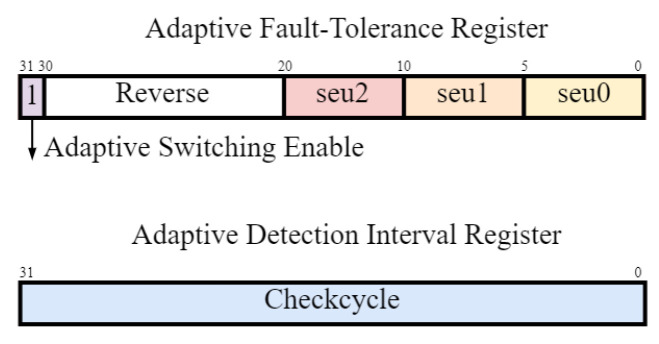
Adaptive fault-tolerance registers.

This mechanism classifies applications according to their fault-tolerance requirements and defines three tiers of fault rates. Figure 4 illustrates the process of determining the work mode in the system. This is achieved by configuring the adaptive fault-tolerance register with the following segments: *seu0*, *seu1*, *seu2*, and *eccu*, and the adaptive detection interval register with the segment: *Checkcycle*. The system functions in a high-performance mode to handle faults falling within the *seu0* and *seu1* range, a balanced mode for faults falling within the *seu1* and *seu2* range, and a high-reliability mode for errors that exceed *seu2*. In high-performance mode, a redundant mode transition is triggered when the error rate in secure storage, as measured by ECC verification, surpasses the *eccu* threshold during the *Checkcycle* interval.

A performance counter has been developed that utilizes the system’s original performance monitoring counter. Redundant mode switching is based on the performance sampling counter and the fault-tolerance register. The former is used when the application has low fault tolerance requirements or inherently possesses a certain level of fault tolerance. In contrast, the latter is used for applications that demand high fault tolerance requirements.

### 3.3. Dynamic Mode Switching

To smoothly transition from high-performance mode to balanced/high-reliability mode or from balanced mode to high-reliability mode, the CPUs must synchronize their states. This can be achieved by having the CPUs save checkpoints to their safety memory ahead of time. The slave CPU/CPUs can then retrieve the checkpoint information from the master CPU’s safety memory and load it into their general and control registers. This checkpoint information includes the PC address that will be assigned to the slave CPUs, allowing the master CPU and slave CPUs to operate in lock-step and stay in sync. When transitioning to a higher-performance mode, there are two different scenarios to consider. In the first scenario, after assisting the master CPU in completing the lock-step operation, the slave CPU/CPUs will resume their previous program execution. At this point, the slave CPU/CPUs will initiate a DMA transfer to restore their previous checkpoint into their registers and control registers. In the second scenario, it is assumed that the slave CPU/CPUs’ previous operational state is no longer relevant. In this case, the system only needs to configure the control registers to enable the slave CPU/CPUs to have independent input and output.

When operating CPUs under different modes, the Input–Output Selection Module is required to select suitable inputs. In balanced mode, CPU1 input is bypassed to CPU2, while CPU3 has its independent input. In high-performance mode, all three processors have their independent inputs and outputs. In high-reliability mode, the output of CPU1, CPU2, and CPU3 is selected by a majority voter.

### 3.4. Cache Mode Switching

This paper proposes an improved cache strategy that reduces the performance losses caused by complete cache flushes. It uses a validity list and a flag list, where the flag is set to valid when the cache line is loaded in high-reliability mode and reset to invalid when loaded or modified in high-performance mode.

**Figure 5:**
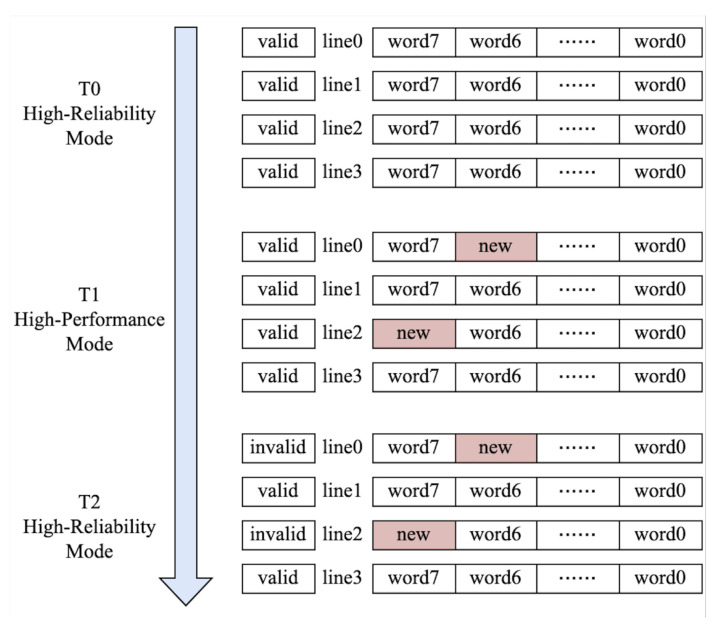
Cache mode-switching state update between different modes.

When transitioning states, it is necessary to consider cache state consistency. The simplest approach is to perform a complete cache flush with each transition from high-performance mode to high-reliability mode or balanced mode. However, this method leads to a reduction in cache utilization, resulting in performance wastage. Cache utilizes a validity list to indicate the validity of each cache line. This paper proposes an improved strategy by introducing a new flag list alongside the existing validity list. The flag is set as valid if the associated cache line is loaded in a secure mode. If the processor loads or modifies a cache line in high-performance mode, this flag is reset to invalid. Utilizing valid cache lines in high-reliability mode can reduce performance losses caused by a complete refresh. The operation is illustrated in Figure 5. At time T0, the system switches from high-reliability mode to high-performance mode. In phase T1, the processor modifies line 0 and line 2 in the cache. The validity bits of these two cache lines are marked in the newly added validity list. At time T2, when the processor switches back from high-performance mode to high-reliability mode, the cache lines modified during the high-performance mode, namely line 0 and line 2, are invalidated, while the remaining cache lines, line 1 and line 3, are still considered valid. This method effectively reduces the performance losses caused by a full cache refresh.

## 4. Redundancy Mode Switchable Multi-Core Processor System

**Figure 6:**
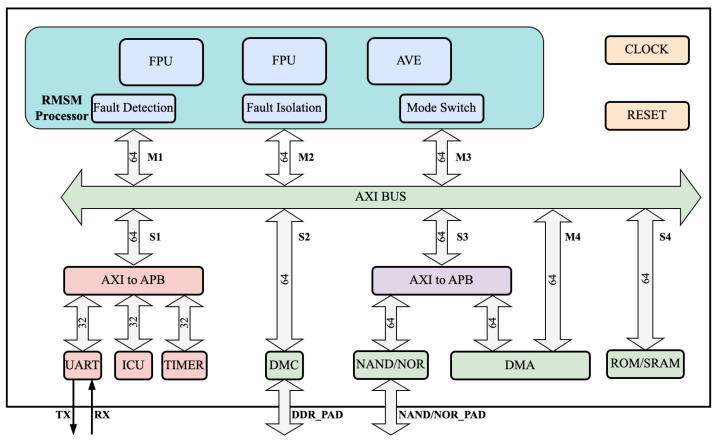
RMSM Processor system structure.

In our work, we have designed a multi-core system that supports a redundancy mode-switchable multi-core processor system architecture. The RMSM processor system designed in this paper includes the RMSM processor and peripheral controller IPs such as DDR, NandFlash, UART, and interrupts. The structure of the RMSM processor system is illustrated in Figure 6. The processor’s external instruction and data interfaces use the OBI (Open Bus Interface) bus. The instruction and data cache front end uses the OBI bus, while the back end uses the AXI bus. The data cache supports configurable write-through/no-write-allocate or write-back/write-allocate strategies. Modules such as fault detection, fault isolation, and mode switching are added outside the core to support lock-step redundancy and mode switchability. Inside the core, checkpoint backup register files and hardware-dedicated DMA are added to the pipeline to support rapid checkpoint backup and recovery.

Three redundancy mode types are defined based on different reliability and performance requirements.

- High-reliability Mode
  The high-reliability mode is a fault-tolerant system that employs TMR. In this mode, the data stream of Processor 1 is fed into Processors 2 and 3. All three processors execute the same program, resulting in a performance equivalent to that of a single-core processor. TMR mode is a type of forward error correction that can mask error outputs caused by soft errors without requiring a checkpoint backup mechanism. It is built on a dual-core lockstep that features triple modular redundancy and adds a multiple-input voting circuit for correct status output.
  System faults can be classified as repairable or non-repairable. If only one processor has an error, it can be restored to normal operation. However, if two or more processors have errors, the system will enter an unrecoverable state, necessitating a reset. The TMR mode, with its robust architecture, offers a high degree of reliability and fault tolerance for mission-critical applications.
- Balanced Mode
  The balanced mode of operation involves Processor 1 and Processor 2 working in a dual-core redundancy mode, while Processor 3 operates independently. This mode of operation aims to balance reliability and performance. The input data stream of Processor 1 is bypassed to Processor 2 after passing through mode selection logic. The output data streams of Processor 1 and Processor 2 are synchronized through a dual-core lock-step before being released. Processor 3 operates with independent input and output data streams. The dual-core lock-step redundancy fault-tolerance method typically employs fault detection and fault recovery techniques. This paper utilizes hardware pipeline lock-step redundancy technology, which encompasses fault detection, fault recovery, and fault isolation at the hardware level. Fault detection combines processor replication with checksum circuits, while fault recovery employs fully hardware-based checkpoint backup and pipeline rollback. Fault isolation prevents errors from propagating to external storage.
- High-performance Mode
  When operating in high-performance mode, the system foregoes fault-tolerance capabilities. Specifically, Processor 1, Processor 2, and Processor 3 operate concurrently to process programs. All three processors maintain independent input and output data streams, each with its own distinct bus, data storage, and instruction storage. Communication between multiple cores is facilitated through shared RAM, allowing each core to trigger an interruption for the others. This communication occurs via data preparation and triggered interruptions, rendering processors in this mode comparable to a standard tri-core processor.

### 4.1. Optimizing for a System with Enhanced Reliability

Improvements have been made to the checkpoint backup mechanism and pipeline rollback method. Compared to the traditional software checkpoint with poor real-time performance and high performance overhead, real-time backup of hardware register files can quickly recover checkpoints within short cycles. To address security concerns related to the backup of register files, a software–hardware coordinated checkpoint-specific DMA backup approach is proposed. This method involves the periodic generation of backup instructions by software, which uses hardware DMA to back up checkpoints to external secure storage without affecting normal processor execution. This significantly reduces the performance overhead associated with checkpoint backup. Additionally, to guarantee reliability while maintaining performance, we introduce a fault isolation method for pipeline processors based on read–write cache flags.

#### 4.1.1. Software-Hardware Coordinated Checkpoint Backup Method

**Figure 7:**
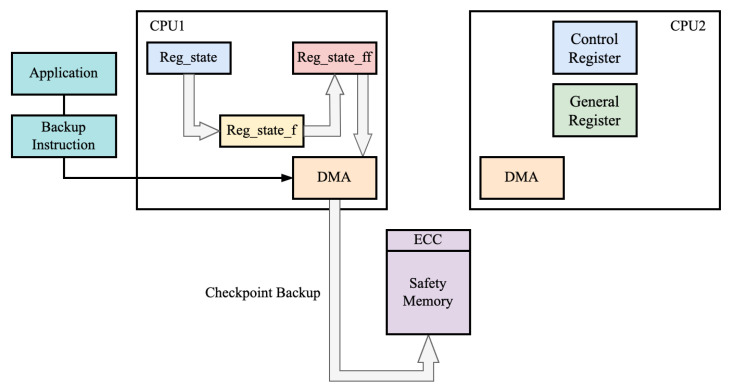
Software–hardware coordinated checkpoint backup.

Both regular and backup registers have the same error rates under harsh workplace conditions. This means that if both the architectural and backup registers experience errors at the same time during execution, the processor will be unable to function correctly, leading to continued errors. To address this issue, a software–hardware coordinated checkpoint backup method has been proposed. This method involves setting periodic backup instructions at the software level to back up correct checkpoints to external memory that is isolated from soft errors. To reduce the backup time, a dedicated checkpoint DMA transfer unit is employed to assist in the backup process. The system architecture is shown in Figure 7. At the software level, specialized checkpoint backup instructions are added. In normal operating conditions, the processor continuously performs automatic hardware checkpoint backups internally. When the processor executes the checkpoint backup instruction, the internal DMA transfer module is activated to transfer the backed-up register checkpoint, reg_ff, to the external safety memory for soft error isolation. At this point, the normal pipeline checkpoint backup is paused, meaning reg_f and reg_ff are no longer updated during the DMA transfer process until it is completed. Additionally, since the reg_ff register files transferred by DMA do not interfere with the processor’s active register files, the processor can continue running during the hardware checkpoint backup process. However, during the hardware DMA checkpoint backup period, the processor loses real-time pipeline register recovery functionality. If an error occurs during this stage, the processor will pause and revert to the state set by the last software checkpoint. By using the hardware-dedicated DMA and software-hardware coordinated checkpoint backup method proposed in this paper, the performance overhead caused by software checkpoint backup and the risk of system deadlock due to reverting to an erroneous state is reduced.

#### 4.1.2. Pipeline Structure for Lock-Step Fast Rollback

**Figure 8:**
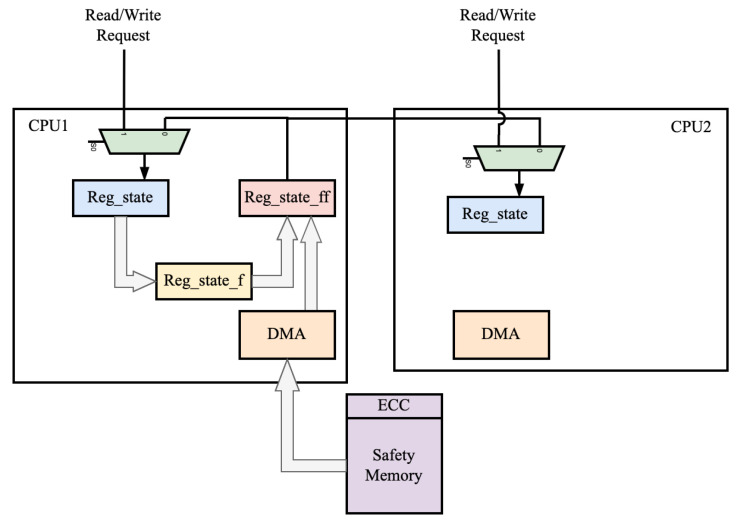
Pipeline rollback process.

There are two recovery schemes for restoring processor register states. Scheme 1 involves direct hardware connecting the backed-up register ‘reg_state_ff’ to the general purpose registers, enabling the restoration of register states within a single clock cycle. Scheme 2 uses an external DMA for state recovery. If the processor detects an error even after recovering from the backup checkpoint register, it triggers the DMA to recover from storage with soft-error isolation. See Figure 8 for an illustration of this process.

Only CPU1 undergoes a hardware-based register checkpoint backup to minimize area overhead. The output is restored to both CPU1 and CPU2 registers. In case of errors, CPU1 reads the correct backup from secure storage to restore the main register file of either CPU1 or CPU2.

#### 4.1.3. Fault Isolation Method for Pipeline Processors Based on Read-Write Cache Flag

**Figure 9:**
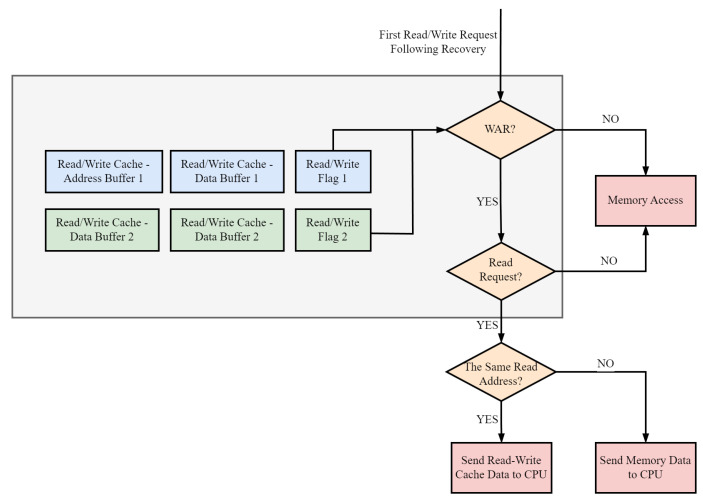
Fault-isolation method based on read–write cache flag for pipeline checkpointing.

This paper introduces a novel solution that addresses the issue of read-after-write dependency during fault isolation. As shown in Figure 9, the proposed solution involves a cache that not only stores write requests but also caches read requests. The cache is designed to store read and write operations initiated by the processor and is accessed by the bus. In the event of a fault, the cache compares the first read request initiated by the processor with the read address in the read–write cache. If the addresses match, the data are directly retrieved from the cache to ensure accuracy.

In an in-order processor’s four-stage pipeline, when executing a memory access instruction, the processor must wait for the external response signal before proceeding to the next instruction. As a result, within the synchronization time of the processor fault detection signal, at most, one write request will be initiated. To overcome the read-after-write dependency issue mentioned earlier with regard to memory access, two address buffers and two data buffers are set up. When the system is operating normally, in addition to sending read and write requests to the bus, information about each read and write request is cached in the buffers. A 1-bit read–write flag is set to represent which type of memory access request is being processed.

After a fault, the processor checks whether the buffer’s read–write flag indicates a read-after-write dependency. If no read-after-write dependency is detected, it means that the processor did not read the newly written value during the synchronization period of the fault detection signal. Subsequently, the processor checks whether the first read and write request initiated after fault recovery is a read request. If it is a read request and the read address matches the read address in buffer 2, it indicates that the processor executed this read request before the fault recovery process, and the correct data are retrieved from data buffer 2. In all other cases, normal access to the storage is achieved, and the read–write cache buffers are updated.

This solution addresses the issue of read-after-write dependency that arises during fault isolation. It enables normal execution on the system bus and allows for interaction with other processors or external devices without any additional synchronization operations required at the software level. The proposed solution is a promising approach for improving the reliability and efficiency of memory access in complex computer systems.

## 5. Evaluation

### 5.1. Set Up

Our work involves evaluating a tri-core system consisting of three RISC-V cores. We propose a dynamic switching strategy in Section 3 and detail a redundancy-mode switchable multi-core processor system in Section 4. Our application scenario is designed for the PV system controller, focusing on controlling the MPPT intelligence algorithm. We use SPEC2017 as the test set instructions, which include integer and floating point operations. We can simulate the event-driven procedure of the PV system, including the real-time response and energy management control strategy of the MPPT algorithm, using the 505.mcf_r benchmark. The search and decision-making processes of AI algorithms in the 531.deepsjeng_r and 541.leela_r benchmarks can be compared to the process of the MPPT algorithm finding the maximum power point. These algorithms demonstrate how to make effective decisions in complex scenarios, similar to the objective of the MPPT algorithm in optimizing electrical output in PV systems. We use the gem5 simulator at the RTL level to evaluate the performance and reliability. In Section 5.2, we introduce a high-efficiency evaluation framework, while the result of reliability evaluation is based on error counter statistics and software calculations of the error rate. We show the resource utilization of an FPGA development board with a multi-core processor clock set to 50 MHz in Table 1.

### 5.2. Soft Error Injection

Soft error injection is a method used to assess the reliability of a system [25]. Soft errors are random errors that can occur at any time or place. The randomness of these errors is mainly caused by two factors: time randomness and spatial randomness. Time randomness refers to the random occurrence of faults during the entire time period from reset to the end of the simulation, when the test processor operating frequency is 500 MHz. Each time a fault occurs, it is considered a random time-discrete point, and this point should follow a uniform probability distribution throughout the entire time period. In order to ensure spatial randomness, all signals inside the processor are extracted, and then a large number of random signal flip errors are generated through a series of random events. These errors are then injected into the system through software.

### 5.3. Evaluation Framework of TMR

**Figure 10:**
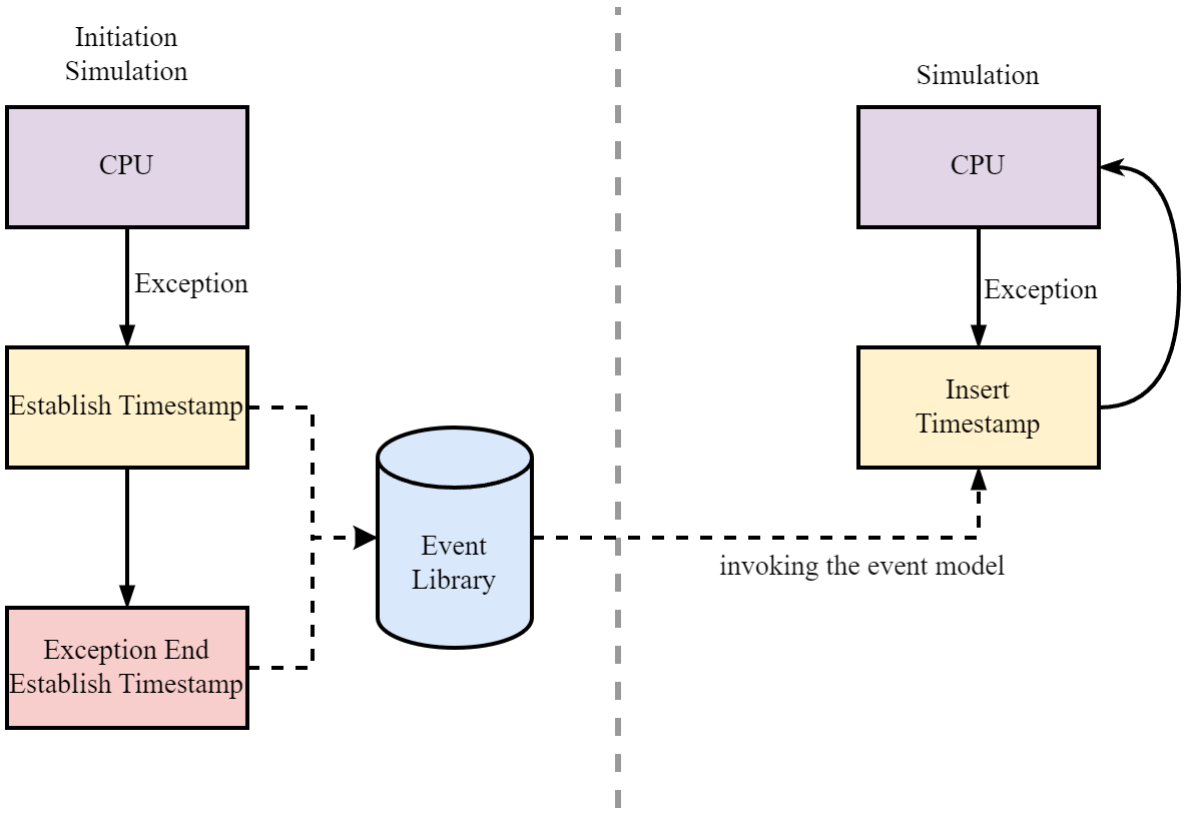
Evaluation framework.

Gem5 is a modular discrete event-driven computer system simulator platform. In its verification environment, the verification results are reactions to parameter settings. Based on this feature, we propose a new verification framework for redundant structures by using a single-core system to verify its overall performance. The specific structure is shown in Figure 10. The normal operation and abnormal states of the single core are separated and simulated as individual events. For example, when a mode switch is required, a switch signal is initiated, followed by pipeline flushing, processor lock-step, context copying, and so on. Therefore, the entire mode-switching process can be considered as a combination of multiple events. We achieve the simulation of mode switching by constructing events and inserting them into the gem5 event queue.

In fault injection, faults that do not propagate and affect the computation results in the hardware structure are called ineffective faults. Since ineffective faults do not affect the CPU’s operation, the single-core verification method and the multi-core verification method are indistinguishable. For effective faults in the multi-core verification process, the following situations may occur: (1) the voting mechanism masks the errors caused by effective faults in the processor; (2) the voting mechanism does not mask the errors caused by effective faults in the processor, and the pipeline is rolled back; (3) effective faults directly cause the CPU with the fault to be unable to operate normally, and that CPU performs a pipeline rollback, waiting for the other CPUs to complete their execution. Although the causes of these situations are different, the solutions after their occurrence can all be achieved by constructing exception events. Therefore, the performance of a multi-core redundant structure can be inferred from the single-core operation results and the construction of multiple event models. This approach effectively utilizes the features of the gem5 simulator, greatly reducing verification overhead and improving verification efficiency.

### 5.4. Results and Analysis

In this section, we start by validating the effectiveness and computational efficiency of the intelligent MPPT algorithm on the RMSM system. Subsequently, we compare the reliability of our proposed architecture with a state-of-the-art Adaptive Lock-Step System for Resilient Multiprocessing Architecture under separate operational modes to demonstrate the superior performance and robustness of the RMSM system. Then, we compare the recovery rates and execution times of the adaptive switching mode against three individual modes to further substantiate the advantages of the RMSM system. To assess the real-world performance of the RMSM system, we use a selection of SPEC2017 benchmarks that simulate practical PV system control scenarios, using an intelligent MPPT algorithm for optimal energy tracking.

#### 5.4.1. Validation of the Intelligent MPPT Algorithm on the RMSM System

**Figure 11:**
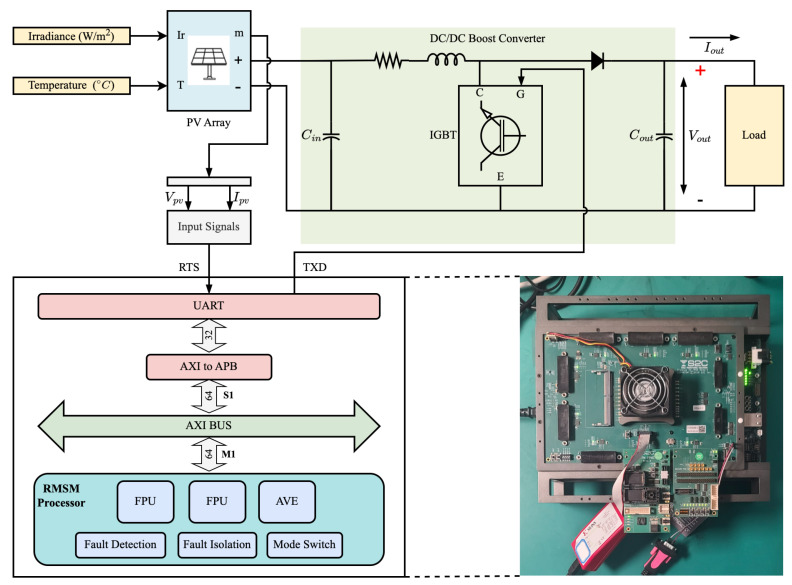
MPPT validation platform.

We use MATLAB 2023b to simulate PV arrays and generate real-time output signals fed into a VU440 FPGA board integrated with RMSM systems for MPPT calculations, as shown in Figure 11.

**Figure 12:**
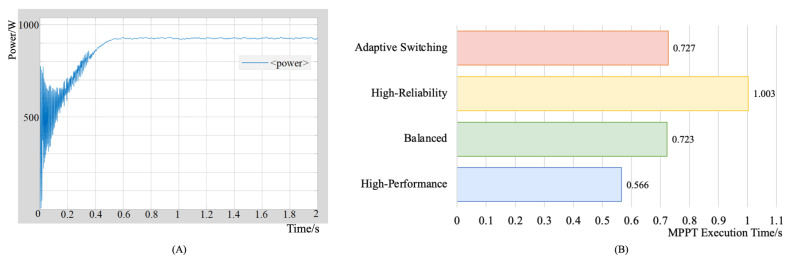
MPPT algorithm execution result. (**A**) Power tracking curve under high-performance mode. (**B**) Execution times of different modes.

This framework effectively simulates the operational efficiency of the RMSM system when implementing the intelligent MPPT algorithm in real-world scenarios. In detail, we use Simulink to create a PV array model that dynamically generates digital current and voltage outputs based on irradiance and temperature. Then, current and voltage are transmitted to the FPGA board via UART serial protocols, enabling real-time data exchange between the MATLAB environment and the FPGA hardware. The RMSM system with a Gray Wolf Optimizer bitstream has been loaded onto the FPAG board in advance. Therefore, the FPGA board can accept current and voltage as input to the Gray Wolf Optimizer to calculate and record the duty cycle, which is used to provide control signals for the DC-DC converter. The recorded duty cycle is sent to a DC-DC boost converter to automatically adjust to the voltage and current corresponding to the maximum power point. We evaluate the performance of the MPPT algorithm in four operating modes, as illustrated in Figure 12. The results demonstrate the effectiveness of the proposed RMSM system in accurately identifying the maximum power point. Among the four modes, the adaptive switching mode exhibits the second-highest performance, completing the algorithm execution in 0.727 s. In the following sections, we will further demonstrate the high performance and reliability of the RMSM system in different scenarios of complex PV systems.

#### 5.4.2. Single-Mode Reliability Analysis

This paper uses the proposed soft error injection method to inject 5000 soft errors into the mcr_f test program. These soft error test cases are randomly generated by a script. The reliability of the system is analyzed under different operation modes on the simulation platform and compared with the reliability result of the Adaptive Lock-Step System for Resilient Multiprocessing Architecture proposed by Chen et al. [20]. The statistical results of the simulation are shown in Table 2. The invalid fault rate (*IFR*) is defined as the proportion of invalid faults in relation to the total number of faults. The failure recovery rate (*FRR*) is defined as the proportion of normally executed test cases to the total number of injected soft error test cases.

(1) $$IFR=\frac{Invalid}{Invalid+Success+Fail}$$

(2) $$FRR=\frac{Success}{Success+Fail}$$

The IFR of our proposed soft error injection method is around 82%, which is slightly lower than that of the fault injection method proposed by Violante et al. [26]. This indicates that our proposed approach is valid and reasonable. In high-performance mode, the system lacks fault tolerance, so valid errors can lead to abnormal execution. In balanced mode, the dual-core part can recover faults using checkpoint backup and rollback. However, the independent core part has no fault tolerance but an ECC check, and failures in this mode are mainly caused by the independent core. In high-reliability mode, the system uses TMR to perform fault tolerance, allowing most soft errors to be tolerated. When we compare it with the FRR associated with Chen et al.’s method, the advantages of our proposed system become evident. In high-performance mode, the proposed system’s FRR is 1.75% lower than that of the single-core mode, mainly due to its reliance on triple-core parallelism, which requires coordination and task management across cores and introduces issues related to shared resource access. In balanced mode, the FRR of our system is 10.92% higher than in the DCLS mode. This improvement is attributed to techniques such as checkpoint backup and rollback, pipeline structures for lock-step fast rollback, and fault isolation methods based on read–write cache flags. The respective figure is relatively the same when comparing the high-reliability mode with the TMR mode: around 99.0%. Due to the error injection method used in the TMR mode injecting 3 bits per injection, additional tests are performed to test multi-bit error injection in the high-reliability mode. The additional tests include 2 bits and 4 bits per injection, and the injection process is repeated 5000 times, respectively. The result of the multi-bit injections is shown in Table 3, the FFRs are all around 99.0%.

#### 5.4.3. Dynamic Switching State System Reliability and Performance Comparison

**Figure 13:**
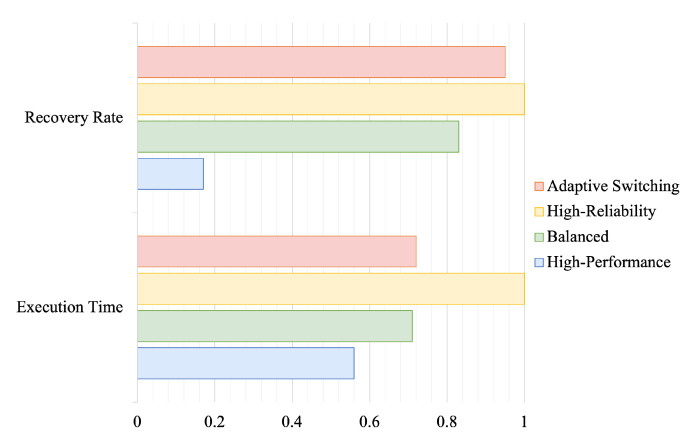
Mode efficiency comparison: adaptive mode versus single-mode systems.

The adaptive dynamics of the recovery system are compared with three single modes, keeping the soft error injection method unchanged. The experimental results are shown in Figure 13. The results show that in the dynamic switching mode, the fault recovery rate decreases compared with the single mode, but remains at a high level. The fault recovery rate decreases compared to that of the single mode but remains at a high level. This is due to the lower fault recovery rate of the dynamic switching mode in high-performance scenarios, which reduces the overall system fault recovery rate. Performance comparisons indicate that system performance remains high in the dynamic switching mode.

#### 5.4.4. Benchmark-Based Simulation of PV System Control

Oriented toward the intelligent MPPT algorithm in the complex PV system under real-world scenarios, we select a set of benchmarks from SPEC2017 to estimate the performance of the RMSM system. Intelligent MPPT algorithms often involve intensive computations, real-time response, optimization processes, and decision-making capabilities. To align with the computational characteristics of intelligent MPPT algorithms, we choose 531.deepsjeng_r, 541.leela_r, 505.mcf_r and 519.lbm_r benchmarks from SPEC2017 to simulate the workload characteristics of MPPT.

- 531.deepsjeng_r benchmark involves extensive use of search algorithms and decision-making logic. Intelligent MPPT algorithms need to explore multiple scenarios to find the maximum power point under various conditions. The search-heavy nature of 531.deepsjeng_r is analogous to the optimization process in MPPT, where it continuously searches for the optimal power output.
- 541.leela_r Benchmark uses Monte Carlo Tree Search and neural network evaluations. Intelligent MPPT algorithms take advantage of machine learning for optimization. The decision-making and search processes in 541.leela_r mimic the MPPT algorithms’ need to adjust to changing environmental conditions to maximize power output, making this model suitable for evaluating AI-based MPPT algorithms.
- 505.mcf_r involves heavy integer computations and optimization algorithms. Similarly to how 505.mcf_r optimizes transportation networks, MPPT algorithms optimize the power output of PV systems. This model tests the system’s ability to handle dynamic optimization and resource allocation, which aligns well with the continuous adjustment needed in MPPT to maximize efficiency.
- 519.lbm_r benchmark requires intensive numerical computations and high memory bandwidth. Intelligent algorithms require high-frequency numerical calculations to adapt to rapidly changing environmental inputs (current, voltage, and irradiance). The dense computational workload of 519.lbm_r is effective in simulating the high computational demands of MPPT algorithms, especially for evaluating the performance of the RMSM system under high-load conditions.

**Figure 14:**
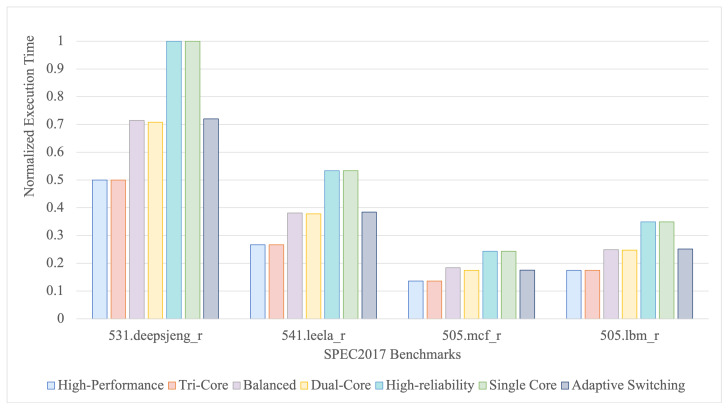
Comparison of the adaptive switching mode and other modes under different benchmarks used to simulate the intelligent MPPT algorithms.

Using these benchmarks, we can effectively simulate the computational, optimization, and real-time response demands of intelligent MPPT algorithms in a PV system. The performance of the proposed RMSM is evaluated by using the gem5 simulator at the RTL level, under both adaptive-switching and single-mode conditions. We compared it against single-core, dual-core, and tri-core configurations without fault tolerance. As shown in Figure 14, the RMSM system demonstrates competitive execution times on benchmarks designed to simulate MPPT algorithms. In particular, it outperforms the high-reliability mode and single-core system without fault tolerance, delivering superior efficiency. Compared with the balanced mode and fault-tolerance-free dual-core system, the RMSM system exhibits similar performance across all benchmarks, except for a slight lag in the 531.deepsjeng_r benchmark. Although the high-performance mode and the fault-tolerance-free tri-core system achieve approximately 40% faster execution times than the RMSM system, our approach offers a significant advantage in terms of reliability, which improves by about 5.58 times. This highlights the superior adaptability and robustness of the RMSM system, especially under dynamic switching conditions, where it effectively balances performance and reliability. By adaptively adjusting core usage and operating mode, the RMSM system achieves near-high performance speeds while substantially enhancing the system’s fault tolerance.

## 6. Conclusions

Current N-modular redundant adaptive switching systems for the MPPT algorithm in PV systems face challenges when it comes to balancing performance and reliability. Specifically, relying solely on software-level reliability detection is less efficient than leveraging hardware for real-time statistical error rate analysis. Additionally, existing approaches often use error rates as the sole criterion for mode switching, leading to frequent transitions that degrade overall system performance. To address these limitations, we propose a novel joint statistical mechanism that incorporates both error rates and performance as switching metrics, enabling efficient adaptive switching tailored to specific application scenarios. This approach not only mitigates soft errors caused by high radiation to ensure substantial reliability but also meets the high computational efficiency demands of photovoltaic systems. Building on this adaptive switching strategy, we developed a redundancy mode-switchable multi-core processor that is designed to dynamically balance efficiency and robustness. Furthermore, optimization techniques were introduced to fine-tune the relationship between performance and reliability, further enhancing the system’s capabilities. The proposed system was rigorously evaluated under various conditions, including real-world scenario simulations and benchmark-based assessments.The experimental results demonstrate an average reliability of 99.2%, showing that our system outperformed the state-of-the-art adaptive lock-step system by 3%. Additionally, the redundancy mode-switchable multi-core processor achieved 1.26 times higher performance compared to TMR systems and 5.58 times higher reliability than tri-core systems. These results underline the system’s potential to significantly improve both computational efficiency and fault tolerance, offering a robust solution for practical PV applications.

## Figures and Tables

**Table 1:** FPGA utilization.

Resources	Resources Occupation
LUT	43,115
LUTRAM	218
FF	32,155
BRAM	558
DSP	12
MMCM	3

**Table 2:** Statistical results of IFR and FFR in different modes.

	Operation Mode	Invalid	Success	Fail	IFR	FRR
Our Work	High-Performance	4114	756	130	82.28%	85.33%
	Balanced	4129	864	7	85.28%	99.20%
	High-Reliability	4163	834	3	83.26%	99.64%
Chen et al. [20]	Single-Core	-	3843	517	-	87.08%
	DCLS	-	7062	938	-	88.28%
	TMR	-	8964	36	-	99.60%

**Table 3:** Statistical results of multi-bit fault injection under triple-modular redundancy.

	The Bit Width per Injection	Invalid	Success	Fail	IFR	FRR
Chen et al. [20]	3	-	8964	36	-	99.60%
Our Work	1	4163	834	3	83.26%	99.64%
	2	3547	1450	3	70.94%	99.79%
	4	784	4178	39	15.68%	99.09%
	8	126	4831	43	2.52%	99.11%

## Data Availability

Data are contained within the article.
